# Postoperative psychogene Hemiparese

**DOI:** 10.1007/s00101-023-01357-2

**Published:** 2023-11-22

**Authors:** Anna Maria Pesch, Axel Schmutz

**Affiliations:** Klinik für Anästhesiologie und Intensivmedizin, Universitätsklinikum Freiburg i. Brsg., Hugstetter Str. 55, 79106 Freiburg, Deutschland

## Anamnese

Ein 33-jähriger Patient stellt sich bei vorausgegangener Schnittverletzung und persistierender Hypästhesie des linken Daumens zur Revisionsoperation vor. Die initiale operative Versorgung erfolgte komplikationslos vier Monate zuvor in Lokalanästhesie.

Bis auf einen Nikotinabusus werden Vorerkrankungen oder Dauermedikation verneint.

## Perioperative Versorgung

Die präoperative Kommunikation auf Deutsch ist trotz eingeschränkter Sprachkenntnisse adäquat möglich. Für die Revisionsoperation erhält der Patient eine totale intravenöse Anästhesie (TIVA) mit Propofol mittels „target controlled infusion“ (TCI, Injectomat™ total intravenous anaesthesia [TIVA] Agilia, Fresenius Kabi, Bad Homburg, Germany) und einer Dosierung zwischen 3 und 5 µg/ml („effect site concentration“, Schnider-Modell). Darüber hinaus erhält der Patient Sufentanil mit einer Gesamtdosis von 75 μg für den 3‑stündigen Eingriff in Blutsperre. Die Atemwegssicherung erfolgt über eine Larynxmaske der zweiten Generation (i-gel®, Intersurgical®Limited, Berkshire, UK). Die Einleitung sowie der intraoperative anästhesiologische Verlauf gestalten sich unauffällig. Der Patient präsentiert sich allzeit normoton (MAD ≥ 65 mm Hg). Postoperativ gelingt dem suffizient spontan atmenden Patienten eigenständig die Umlagerung vom OP-Tisch zurück ins Bett.

Etwa eine Stunde nach Ankunft im Aufwachraum präsentiert sich der Patient mit starrem Blick und inadäquater, klagender Kommunikation auf Kurdisch. Eine i.v.-Analgesie mit Piritramid (kumulativ 6 mg) zeigt keine Verbesserung.

Der Patient ist hämodynamisch stabil, er liegt mit starrem Blick im Bett und nimmt bei direkter Ansprache nur sehr verlangsamt Blickkontakt auf. Die Pupillen sind beidseits mittelweit, isokor und lichtreagibel. Über eine venöse BGA werden eine Elektrolytentgleisung und Hypoglykämie ausgeschlossen.

Bei Somnolenz und dem Fehlen weiterer wegweisender Symptome wird probatorisch Physostigmin (10 mg fraktioniert i.v.) verabreicht um ein eventuell vorliegendes zentrales anticholinerges Syndrom (ZAS) zu behandeln. Die Symptome verbessern sich daraufhin nicht.

Die Symptomatik einer linksseitigen Hemiparese in Kombination mit einer Sprachstörung lässt sich keinem vaskulärem Versorgungsgebiet zuordnen. Es wird konsiliarisch ein Neurologe hinzugezogen (Infobox 1).

### Infobox Fachneurologischer Untersuchungsbefund im Aufwachraum

„Befund ca. 16 Uhr: wach, kaum kooperativ für Auff, spricht (klagend) nur immer wieder die gleichen Worte auf Arabisch/Türkisch, kein Neglekt. Kein Meningismus. Hirnnerven: Pupillen mw, isokor, LR +/+, Gesichtsfelder schreckreaktiv frei, keine Blickparese/Ptosis/path. Nystagmen, glatte Blickfolge, keine Parese. Motorik: nach passivem Anheben re Arm und Bein etwas gehalten, li sofortiges schlaffes Runterfallen. Arm fällt, über dem Gesicht gehalten, allerdings auf den Thorax, schlaffe HP li dennoch nicht ausschließbar. Babinski beidseits negativ. Sensibilität: Sz-Reiz links scheinbar weniger wahrgenommen.“

## Diagnostik

- BGA venös:
  - pH 7,358; pCO_2_ 41,2 mm Hg; pO_2_ 73,6 mm Hg; Sauerstoffsättigung 90,1 %; ctO_2_ 18,0 Vol.-%; SBC 22,4 mmol/l; SBE − 2,0 mmol/l; CO-Hb 1,0 %; Met-Hb 0,8 %; O_2_-Hb 88,5 %; Hb 14,4 g/dl; HKT (berechnet) 44,2 %; Natrium 138 mmol/l; Kalium 3,6 mmol/l; Kalzium (ionisiert) 1,19 mmol/l; Chlorid 109 mmol/l; Lactat 0,7 mmol/l; Glucose 88 mg/dl.
- Multimodale Computertomographie (CT):
  - In der nativen CT sowie CT-Angiographie und CT-Perfusion des Schädels zeigt sich kein Hinweis auf eine akute Ischämie oder intrakranielle Blutung. Beim Patienten liegen keine höhergradigen arteriellen oder venösen Gefäßverengungen oder -verschlüsse sowie kein Perfusionsdefizit vor.

## Verlauf

Nach Sichtung der zerebralen Bildgebung ohne pathologischen Befund wird durch den Neurologen der Verdacht auf eine funktionelle Sprachstörung und funktionelle linksseitige Hemiparese a. e. auf dem Boden einer Belastungsreaktion gestellt.

Der Patient verbleibt zur weiteren Überwachung im Aufwachraum. Auch 12 Stunden nach dem Eingriff ist er nicht orientiert, weiterhin verlangsamt und die linksseitige Hemiparese besteht unverändert. Nach 19 Stunden wird eine deutliche Besserung seines Zustands beschrieben. Der Patient ist nun zur Person orientiert; die linksseitige Motorik und Sensibilität sind eingeschränkt vorhanden. Nach Zureden kann eine vermehrte linksseitige Mobilität beobachtet werden. Es erfolgt die Verlegung auf die Normalstation der Handchirurgie. Hier beobachtet auch die Physio- und Ergotherapeutin eine eingeschränkte linksseitige Mobilität.

Am dritten postoperativen Tag erfolgt nach weiterer Besserung der Symptomatik die Entlassung des Patienten ins häusliche Umfeld. Im abschließenden Gespräch mit dem Neurologen lässt sich eruieren, dass die Mutter des Patienten nach der Entwicklung einer Hemiparese und Einlieferung ins Krankenhaus verstarb. Es erfolgt die ausdrückliche Empfehlung einer Psychotherapie um die traumatisierenden Ereignisse aufzuarbeiten.

## Diskussion

Bei postoperativen Bewusstseinsstörungen müssen organische Differenzialdiagnosen wie das ZAS, Elektrolytstörungen oder Blutzuckerentgleisungen, (post-)iktale Phänomene sowie ein zerebrovaskuläres Ereignis ausgeschlossen werden [1, 2]. Jedes akute neurologische Defizit sollte im Zweifel wie ein Schlaganfall behandelt werden. Dies erfordert eine umgehende Bildgebung und die Beurteilung durch einen in der Schlaganfallbehandlung erfahrenen Arzt. Als Differenzialdiagnose bei fehlendem organischem Korrelat sollte auch die Diagnose einer funktionalen neurologischen Störung (FND) in Betracht gezogen werden [1, 3, 4].

Die Prävalenz der FND wird auf 0,5 % in der allgemeinen Bevölkerung geschätzt [5]. Die perioperative Prävalenz ist nicht bekannt [6]. Zu den Risikofaktoren gehören ein Alter von 10 bis 35 Jahren, psychische Vorerkrankungen sowie eine vorausgegangene Episode einer FND. Außerdem wird eine anästhesiologische Assoziation aufgrund möglicher dissoziativer Wirkung der Anästhetika diskutiert. Auch der mit der Narkose einhergehende Kontrollverlust kann eine FND begünstigen [7]. Bei vielen Patienten treten die neurologischen Symptome mit einer Latenz zur Narkoseausleitung auf [4]. Das häufigste Symptom ist der Verlust der motorischen oder sensorischen Funktion einer oder mehrerer Extremitäten [7]. Eine FND kann sich allerdings auch als Bewusstseinsstörung oder psychogener nichtepileptischer Anfall (PNEA) präsentieren [4]. Ein PNEA kann vom epileptischen Anfall durch klinische Beobachtungen wie z. B. die Länge der konvulsiven Phase (i. d. R. ≤ 2 min) oder das Zukneifen der Augen bei der Bulbusinspektion differenziert werden [8]. Bedside-Tests können auch genutzt werden um eine psychogene Ätiologie einer motorischen oder sensorischen Funktionsstörung nahezulegen (Tab. 1 [4, 7]).

**Table 1:** 

Klinisches Zeichen	Durchführung
Abduktor-Zeichen (Bein/Finger)	Die Abduktion der nichtparetischen Seite gegen Widerstand führt zu einer synkinetischen Abduktion der betroffenen Seite
„Hand-Raise drop test“	Der Untersucher lässt den Arm des Patienten über dessen Gesicht fallen. Der Patient vermeidet das Herabfallen des Arms auf sein Gesicht
Hoover-Zeichen	Prinzip der synergistischen Kontraktion. Während der Flexion des nichtparetischen Beins kommt es zu einer unbewussten Extension des zuvor paretischen Beins
Mittellinien-Splitting	Gestörte Wahrnehmung der Sensibilität, die genau durch die Medianlinie begrenzt wird. Kutane Nervenäste der Interkostalnerven überlappen sich mit denen der kontralateralen Seite, sodass ein organischer Sensibilitätsverlust ca. 1–2 cm hinter der Medianlinie zu erwarten ist
„Spinal Injuries Center test“	1. Aufforderung zur aktiven Flexion im Kniegelenk. – nicht möglich 2. Passive Flexion im Kniegelenk, die Füße sind vollständig auf der Unterlage positioniert – selbstständiges Halten der Position vom Patienten möglich

## Fazit für die Praxis

Bei postoperativen akuten neurologischen Defiziten sollte nach Ausschluss der oben genannten organischen Differenzialdiagnosen bei nichtsomatotopischer Symptomkonstellation und positiven klinischen Zeichen für eine psychogene Funktionsstörung eine FND als Differenzialdiagnose ins Auge gefasst werden.
